# Mobile phone virtual reality game for pediatric home burn dressing pain management: a randomized feasibility clinical trial

**DOI:** 10.1186/s40814-022-01150-9

**Published:** 2022-08-18

**Authors:** Megan Armstrong, Jonathan Lun, Jonathan I. Groner, Rajan K. Thakkar, Renata Fabia, Dana Noffsinger, Ai Ni, Rohali Keesari, Henry Xiang

**Affiliations:** 1 Center for Pediatric Trauma Research, The Abigail Wexner Research Institute at Nationwide Children’s Hospital, 700 Children’s Drive, Columbus, OH 43205 USA; 2 Center for Injury Research and Policy, The Abigail Wexner Research Institute at Nationwide Children’s Hospital, 700 Children’s Drive, Columbus, OH 43205 USA; 3 College of Medicine, The Ohio State University, 370 West 9th Avenue, Columbus, OH 43210 USA; 4 Department of Pediatric Surgery, Nationwide Children’s Hospital, 700 Children’s Drive, Columbus, OH 43205 USA; 5 Department of Pediatrics, The Ohio State University, 370 West 9th Avenue, Columbus, OH 43210 USA; 6 Division of Biostatistics, The Ohio State University College of Public Health, 1841 Neil Avenue, Columbus, OH 43210 USA; 7 Pediatric Biostatistics Core, Department of Pediatrics, Emory University School of Medicine, 2015 Uppergate Road, Atlanta, GA 30322 USA

**Keywords:** Virtual reality, Burn, Injury, Pain management, Pediatrics

## Abstract

**Background:**

Virtual reality (VR) gaming is considered a safe and effective alternative to standard pain alleviation in the hospital. This study addressed the potential effectiveness and feasibility of a VR game that was developed by our research team for repeated at-home burn dressing changes.

**Methods:**

A randomized clinical trial was conducted among patients recruited from the outpatient burn clinic of a large American Burn Association–verified pediatric burn center between September 2019 and June 2021. We included English-speaking burn patients aged 5–17 years old requiring daily dressing changes for at least 1 week after first outpatient dressing change. One group played an interactive VR game during dressing changes, while the other utilized standard distraction techniques available in the home for up to a week. Both child and caretaker were asked to assess perceived pain on a numerical rating scale (NRS) of 0–10. For the VR group, patients were also asked to rate various aspects of the VR game on a NRS of 0–10 and caregivers were asked questions assessing ease of use.

**Results:**

A total of 35 children were recruited for this study with 24 fully completing study measures. The majority of participants were male (*n*=19, 54.3%), White (*n*=29, 82.9%), and with second degree burns (*n*=32, 91.4%). Children and caregivers in the VR group reported less pain than the control group at the 4th dressing change. Participants in the VR group showed a clinically meaningful (≥30%) reduction in child-reported overall pain (33.3%) and caregiver-reported worst pain (31.6%) in comparison with subjects in the control group. Children’s satisfaction with the VR remained at a high level across dressing changes over the 1-week period, with reported realism and engagement increasing over time. Over half of the children (54.5%) enjoyed playing the game and did not report any challenges nor any side effects.

**Conclusions:**

Subjects found the VR to be a useful distraction during home dressing changes and reported no challenges/side effects. VR should be considered as a nonpharmacologic companion for pain management during at-home burn dressing changes.

**Trial registration:**

ClinicalTrials.gov Identifier: NCT04548635. Registered September 14, 2020—retrospectively registered

**Supplementary Information:**

The online version contains supplementary material available at 10.1186/s40814-022-01150-9.

## Key messages regarding feasibility

- Virtual reality has been shown to be a useful distraction mechanism for inpatient burn injuries, but there is no research indicating if it is still effective and useful at home and whether the device is easy for caregivers to implement.
- Children and caregivers found the virtual reality to be fun and easy to implement and it was effective at reducing pain.
- Our study established the study design to be feasible and provided important considerations for a future full-scale randomized clinical trial study.

## Introduction

National statistics reported that over 250,000 US children (0–17 years) suffer burn injuries every year [1] and the Centers for Disease Control and Prevention reported in 2019 that burns are the fourth leading cause of death due to unintentional injury in children ages 1–14 years [2]. Over half of pediatric burn injuries seen in the US emergency departments (EDs) are serious enough to merit referral to a burn center according to the US and international guidelines [3, 4]. After being discharged from medical burn care facilities, at-home repeated burn dressing changes are often needed for 2–3 weeks. These dressing changes have been identified by pediatric patients as very painful, with opioid and anxiety medications often being prescribed [5, 6]. Furthermore, the pain experienced during burn dressing changes may cause distress to not only pediatric patients but also their caregivers [7] and this painful experience can serve as a stressor that significantly impacts patients’ post-injury health outcomes [8, 9].

The latest research shows that repeated use of opioid medication for acute pain management is likely to increase the risk of long-term opioid use and risk of opioid addictions [10, 11]. The medical community in the US is diligently working to find the right balance between the risk of undertreating pain and causing unneeded suffering [5, 6] and the risk of over (or inappropriate) prescription of opioids [12]. Nonpharmacologic alternatives have risen to the forefront of pain management research. Methods such as hypnosis, cognitive behavioral therapy, and distraction are now standard protocol in some hospitals [3]. Multiple studies have demonstrated that virtual reality (VR) distraction is clinically beneficial when compared with the current standard of care and provides a much more immersive distraction than standard techniques, such as muscle relaxation and toys [13–15]. In addition to their efficacy, VR games are also rated by patients as enjoyable, user friendly, and having no or minor side effects [13]. VR as a pain alleviation tool for burn injuries is well-studied in the hospital setting; however, there is little literature focusing on its use during at-home dressing changes. Furthermore, previous studies investigating VR gaming for pain management dated back to 1980 and many of them used the bulky computer-based systems, which is not very practical for clinical implementation as well as at-home burn care.

Prior research confirmed that VR provides three unique advantages over traditional nonpharmacological interventions for pediatric burn patients [13, 16]. First, VR technology can create a three-dimensional immersive virtual environment (e.g., visual, auditory, interaction) for actively engaging the pediatric patients’ attention in order to successfully interrupt the pain perception route, consistent with the cognitive-affective model of pain [17, 18] and underlying mechanisms of pain management. The unique, highly immersive experience of presence, interactivity, and embodiment offered by VR-based pain management is therefore distinct from and advantageous to common forms of distraction (i.e., bubbles, books, toys), passively watching television or movies, and playing a two-dimensional handheld video game or game console. Second, because the entire distraction process takes place within a safe, controlled, automated virtual environment [19], VR-based pain management can be safely implemented in home settings. Third, previous researchers have developed VR-based pain distraction with burn patients in mind (e.g., snow world for burn dressing pain management) and preliminary positive results from pediatric and adult patient populations were published. Prior-generation computer-based VR pain distractions required large equipment costs as well as equipment setup and cleaning that required professional training, posing significant obstacles for the VR to be widely adopted in home settings [20]. However, thanks to recent advancements in VR technology, VR-based pain distraction has evolved from expensive and cumbersome pieces of equipment to affordable, lightweight, mobile devices with sizes comparable to a smartphone. This reduction in the size and cost of smartphone-based VR games, coupled with significantly improved system stability, and importantly, accessibility [21], has opened the door to using VR widely for burn dressing pain management in home settings.

Recent meta-analysis and reviews of published studies in the past three decades have provided evidence that VR can effectively distract patients to reduce pain and anxiety across many settings [16, 22–25]. However, prior studies have not investigated the feasibility and barriers to implementing VR games for pain management during at-home burn dressing changes. Feasibility can be “defined as the extent to which a new treatment, or an innovation, can be successfully used or carried out within a given agency or setting,” while acceptability is defined as “perception among implementation stakeholders that a given treatment, service, practice, or innovation is agreeable, palatable, or satisfactory” [26]. Furthermore, almost all the existing studies used computer-based VR that is technologically and financially inaccessible to patient families for everyday use in the home. This study was planned to address the question of whether VR can be an effective tool for pain management during burn dressing changes performed in the home. Based on our prior research, we hypothesized that VR would significantly reduce child- and caregiver-reported pain and that the VR system would be easy to implement in the home setting. In order to address gaps in previous research, our study aimed to (1) examine the effect of VR pain alleviation tool (VR-PAT) on reducing pediatric burn patients’ perceived pain during at-home dressing changes, (2) examine the effect of VR-PAT on reducing pediatric patients’ perceived pain during repeated home burn dressing changes, and (3) examine the wide usability and feasibility of VR-PAT during pediatric dressing changes in a home setting.

## Methods

This randomized clinical trial (RCT) used mixed-methods and multiple sources to test whether a smartphone VR-PAT could feasibly and safely reduce pain during repeated pediatric burn dressing changes at home. We also qualitatively assessed the ease of setup and enjoyment of the program. Based on our prior outpatient clinic research and burn patient numbers, we planned to recruit a total of *n*=40 subjects (*n*=20 per group) for this study. From September 1, 2019, to May 30, 2021 (the end of the funding period), 35 patients were recruited from the outpatient burn clinic of an American Burn Association (ABA)-verified US pediatric burn center and randomly assigned to either the VR group or the control group which used standard home distraction techniques. Inclusion criteria were (1) pediatric burn patients (5–17 years) who were receiving their first outpatient dressing change at our outpatient burn clinic, (2) have a dressing that requires daily changes at home for at least 1 week, and (3) can communicate orally. Exclusion criteria include (1) any wounds that may interfere with study procedures; (2) vision, hearing, or cognitive/motor impairments preventing valid administration of study measures; (3) history of motion sickness, seizure disorder, dizziness, or migraine headaches precipitated by visual auras; (4) minors in foster care; (5) suspected child abuse; (6) unable to communicate in English; or (7) families who do not have access to a smartphone (due to the VR-PAT game requirement). The institutional review board of the Nationwide Children’s Hospital reviewed and approved this study. Written informed consent (and assent for children aged 9 years and older) was collected. This study followed the Consolidated Standards of Reporting Trials (CONSORT) reporting guidelines—extension to randomized pilot and feasibility trials (Additional file 1).

**Fig. 1:**
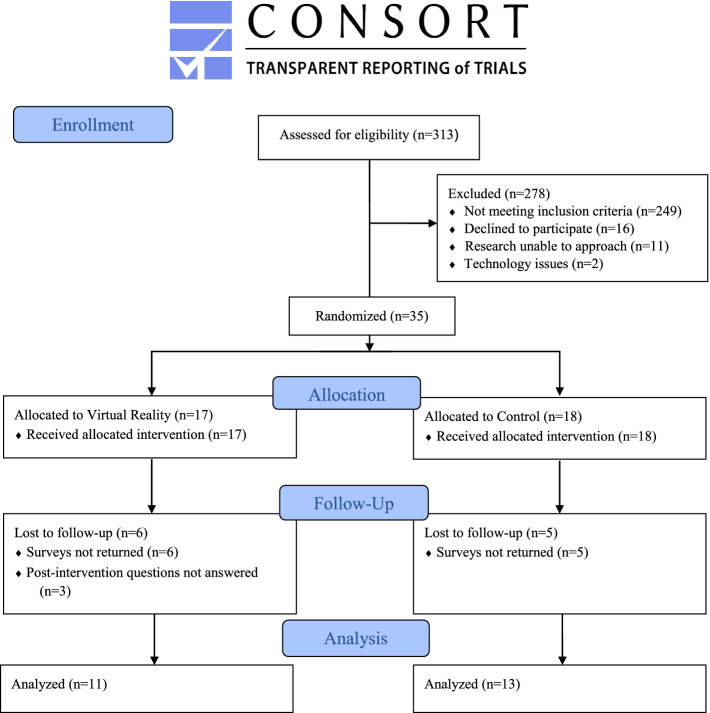
CONSORT flow diagram of participant recruitment

Over the study period, 313 patients were screened, 145 patients were eligible for all factors before knowing the dressing type, and 65 of those patients met our eligibility criteria after learning the dressing type. A trained researcher approached 49 patients for participation and 35 patients consented/assented to this RCT. Of those recruited, 24 participants returned their surveys and completed the study. No other subjects were excluded from the study following recruitment (Fig. 1).

### Study procedures

Potential participants were identified via medical record review and approached in the outpatient burn clinic by a trained researcher. Following informed consent and assent, participants were asked baseline questions about their experience playing video games (days per week playing VR, console, or computer games) before being randomly assigned to either the VR-PAT or a standard of care control group using a 1:1 allocation ratio. A simple randomization method was utilized and built using the random number generator function within Excel. The randomization scheme was uploaded to a Research Electronic Data Capture (REDCap) site [27, 28], and subjects were assigned to a group after pressing the randomization button within the database. The researcher recruiting the subject was blinded to the randomization sequence. Every participant in the study was given a VR headset to bring home, and the control group was instructed to complete the first week of dressing changes without the VR device. Participants and guardians were offered up to eight surveys, to be completed each time a dressing change was necessary for up to 1 week and these surveys were then returned by mail in a pre-paid, self-addressed envelope.

### Child surveys (self-reported)

Participants self-rated overall pain, worst pain, and time spent thinking about pain on a numerical rating scale (NRS) of 0–10 (higher score means more pain). This scale was chosen as it is commonly used to clinically assess pain in the US health care setting and by pain researchers, so we felt subjects would be familiar with and more comfortable answering questions using this scale. It has also been shown to be a quick and appropriate measure for children in this age range [29, 30]. Patients in the VR group were also asked to rate their happiness, fun, engagement, and realism of the game on a NRS of 0–10 (higher score means more helpful). They were also asked to report if the game made them feel not well (side effects).

### Guardian surveys (proxy-reports)

Guardians were also asked to report the participants overall pain and worst pain on a NRS of 0–10 (higher score means more pain). Those in the VR group were asked to report time spent using the VR-PAT, whether the participant declined to use the VR, number of voluntary interruptions, whether the device was helpful and easy to use, and any pain medications (including dose) used for the burn.

Finally, participants in the VR group were contacted after a week of home dressings to ask post-intervention questions about what they liked about the game, did not like about the game, and whether any part of the game or set-up was too hard.

Demographic information was pulled from the electronic medical record. This included date of birth, gender, race, ethnicity, burn date, visit date, percent total body surface area (TBSA), burn severity (1st, 2nd, or 3rd degree), and body area burned.

### Interventions

#### VR-PAT group

Our VR-PAT consisted of a lightweight VR headset with a Virtual River Cruise game that is played on a smartphone. In our pilot study [31], we found that active VR (interacting with VR game) was significantly more beneficial than passive VR (watching the same VR game). Due to these findings, only active VR was used for our intervention group for this study. VR-PAT is a standalone game developed by the Research Information Solutions and Innovation department at Nationwide Children’s Hospital and could be downloaded onto participants’ smartphones using either a QR code or a dedicated website. More information about the specific game can be found in our prior publication [31].

#### Control group

Our standard of care group was able to use any distraction available in the home, including toys, mobile phone, and books. Subjects in the control group were asked not to use the VR device during the first week of dressing changes but encouraged to use it for any dressing following the study week.

### Study outcomes and confounding variables

#### Primary outcome

Our primary outcome was pain associated with burn dressing changes. Pain scores were compared to subsequent surveys over the following week during repeated dressing changes. Secondary outcomes were time spent thinking about pain and caregiver-reported pain, both rated on a NRS of 0–10. We also provided an opportunity for user feedback on VR’s potential effectiveness and areas for improvement.

#### Exploratory outcome

Both children and caretakers were asked to describe the perceived enjoyability and potential adverse effects of the VR. Additionally, patients were asked questions about prior experience with VR and other gaming systems.

### Statistical analysis

Demographic and burn characteristics were described using frequencies and percentages for the categorical variables and means and standard deviation (SD) for continuous variables. Mean, SD, and median were calculated for the primary outcome of reported pain (worst pain, overall pain, and time spent thinking of pain) across dressing changes. Child satisfaction (realism, engagement, happiness, and fun) was calculated as a mean across dressing changes. Qualitative data was collected at follow-up on the child’s utilization experience. Responses naturally fell into some common themes, so data was analyzed using simple counts and reported as frequencies and percentages. Area under the curve (AUC) for child- and caregiver-reported overall and worst pain was calculated by averaging the pain score for each time point, then using the average scores for the control and VR group to calculate the AUC. Since the AUC was calculated based on plots of overall/worst pain score over time using the trapezoid rule, the AUC can exceed 1, depending on the scale of the pain score. AUC can be interpreted as the cumulative exposure to pain over time, and this recent statistical advancement based on AUC methods allows for combining pain intensity score and medication consumption to enhance pain outcomes assessment [32–35], which is innovative and has many pain and opioid consumption assessment research implications. Percent change was calculated between the control and VR group to determine whether pain reduction was clinically meaningful (≥30%) [36–38]. Statistical significance tests were not conducted, and *p* values not reported due to feasibility study design and the small sample sizes in each group. Data analyses were conducted in SAS version 9.4 (SAS Institute).

## Results

The majority of children recruited into this study were White (*n*=29) and male (*n*=19), making up 88.2% and 64.7% of the VR group and 77.8% and 44.4% of the control group, respectively (Table 1). Participants in the control group were slightly older, having a mean of 12.3 years compared to 10.7 years in the VR group. Both groups had small burns (median 1% TBSA), and 16 participants in each group had a 2nd degree burn. Participants in both groups played console and computer games before but had little to no experience with VR games.

**Table 1:** Demographics, burn characteristics, and experience with games of study participants

Characteristics	Intervention group	
	VR (*n*=17)	Control (*n*=18)
**Demographics**		
Gender, *n* (%)		
Male	11 (64.7)	8 (44.4)
Female	6 (35.3)	10 (55.6)
Race, *n* (%)		
White	15 (88.2)	14 (77.8)
Black	2 (11.8)	3 (16.7)
Other	0 (0)	1 (5.6)
Age in years, mean (SD)	10.7 (2.9)	12.3 (3.3)
**Burn characteristics**		
Burn degree, *n* (%)		
Second	16 (94.1)	16 (88.9)
Third	1 (5.9)	2 (11.1)
TBSA (%), median (IQR)	1 (1 - 2)	1 (0.5 - 1.5)
**Experience with games, median (IQR)**^a^		
VR weekly	0 (0 - 0)	0 (0 - 0)
Console weekly	2 (0 - 5.5)	2 (0 - 7)
Computer weekly	7 (2 - 7)	5 (2 - 7)

*Abbreviations*: *TBSA* Total body surface area, *VR* Virtual reality, *n* Frequency, *SD* Standard deviation, *IQR* Inter-quartile range

^a^Days per week playing games on VR, console (i.e., PlayStation®, Xbox, Nintendo Switch^TM^), or computer (including mobile platforms)

Of the 24 subjects who returned surveys, 11 were in the VR group and 13 were in the control group. There were 2 subjects who did not return medication surveys (Table 2). More subjects in the VR group reported using pain medications for the burn injury in dressings 1–5 than subjects in the control group, but did not use any medications after the 5th dressing. Of all the medications used, the vast majority were over the counter medications such as acetaminophen or ibuprofen.

**Table 2:** Reported pain medication use by dressing number and intervention group

Dressing Number	VR, *N* (%)				Control, *N* (%)			
	Total	No	Yes	Missing	Total	No	Yes	Missing
1	11	3 (27.3)	6 (54.6)	2 (18.2)	13	10 (76.9)	3 (23.1)	0 (0.0)
2	11	4 (36.4)	5 (45.5)	2 (18.2)	13	10 (76.9)	3 (23.1)	0 (0.0)
3	9	4 (44.4)	4 (44.4)	1 (11.1)	13	11 (84.6)	2 (15.4)	0 (0.0)
4	8	5 (62.5)	2 (25.0)	1 (12.5)	13	10 (76.9)	3 (23.1)	0 (0.0)
5	8	6 (75.0)	1 (12.5)	1 (12.5)	13	12 (92.3)	1 (7.7)	0 (0.0)
6	5	5 (100.0)	0 (0.0)	0 (0.0)	11	9 (81.8)	2 (18.2)	0 (0.0)
7	5	5 (100.0)	0 (0.0)	0 (0.0)	8	7 (87.5)	1 (12.5)	0 (0.0)
8	4	3 (75.0)	0 (0.0)	1 (25.0)	6	5 (83.3)	1 (16.7)	0 (0.0)

Only 1 subject used opioid medications. All other reported medications were either Acetaminophen or Ibuprofen

**Fig. 2:**
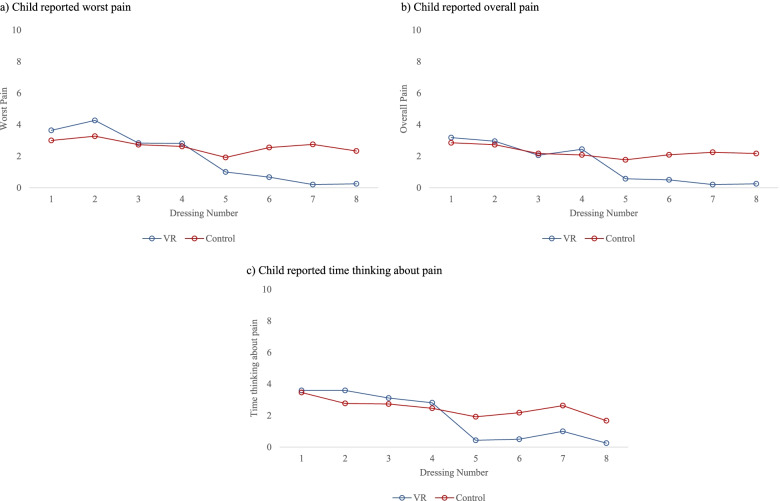
Child reported pain by dressing and intervention. **a** Worst pain, **b** overall pain, and **c** time thinking about pain

Subjects in the VR group completed at least 2 dressing changes while subjects in the control group completed at least 5 dressing changes (Fig. 2). In the VR group, the mean child reported worst pain ranged from 3.6 (SD 2.7) at the 1st dressing to 0.3 (SD 0.5) at the 8th dressing and in the control group, and the range was 3.0 (SD 2.6) at the 1st dressing to 2.3 (SD 2.7) at the 8th dressing. Overall pain ranged from mean 3.2 (SD 2.4) at the 1st dressing to 0.3 (SD 0.5) at the 8th dressing in the VR group and 2.9 (SD 2.3) at the 1st dressing to 2.2 (SD 2.4) at the 8th dressing. The mean time spent thinking about pain ranged from 3.6 (SD 4.1) at the 1st dressing to 0.3 (SD 0.3) at the 8th dressing in the VR group and 3.5 (SD 3.6) at the 1st dressing to 1.7 (SD 2.1) at the 8th dressing in the control group. Children in the VR group reported less pain following the 4th dressing across worst pain, overall pain, and time spent thinking about pain.

**Fig. 3:**
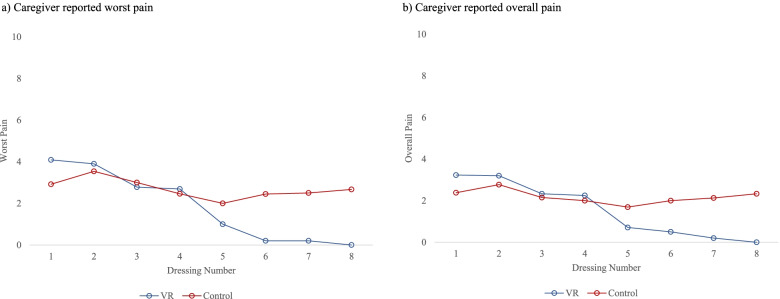
Caregiver-reported pain by dressing and intervention. **a** Worst pain and **b** overall pain

Caregiver-reported pain followed a similar trend as the child reported pain (Fig. 3). In the VR group, the mean caregiver-reported worst pain ranged from 4.1 (SD 3.0) at the 1st dressing to 0.0 (SD 0.0) at the 8th dressing and in the control group; the range was 2.9 (SD 2.8) at the 1st dressing to 2.7 (SD 3.0) at the 8th dressing. Overall pain ranged from mean 3.2 (SD 2.6) at the 1st dressing to 0.0 (SD 0.0) at the 8th dressing in the VR group and 2.4 (SD 2.1) at the 1st dressing to 2.3 (SD 2.6) at the 8th dressing in the control group. Caregivers in the VR group also reported less pain following the 4th dressing across worst pain and overall pain.

**Fig. 4:**
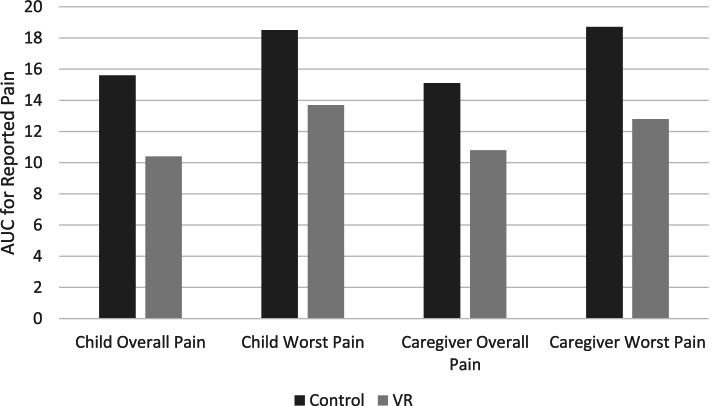
AUC for child and parent reported overall and worst pain scores by intervention group

The AUC for the control group was higher than the VR group for both child- and caregiver-reported overall and worst pain (Fig. 4). The % decrease in child reported overall pain (33.3%) and caregiver-reported worst pain (31.6%) between the control group and VR group reached a clinically meaningful reduction in pain (Table 3). The % decrease in child reported worst pain (25.9%) and caregiver-reported overall pain (28.5%) neared the 30% clinical meaningful threshold.

**Table 3:** Percent change of AUC for child- and caregiver-reported overall and worst pain scores (NRS) by intervention group

	Control	VR	% Change
**Child-reported**			
Overall pain	15.6	10.4	33.3%
Worst pain	18.5	13.7	25.9%
**Caregiver-reported**			
Overall pain	15.1	10.8	28.5%
Worst pain	18.7	12.8	31.6%

**Fig. 5:**
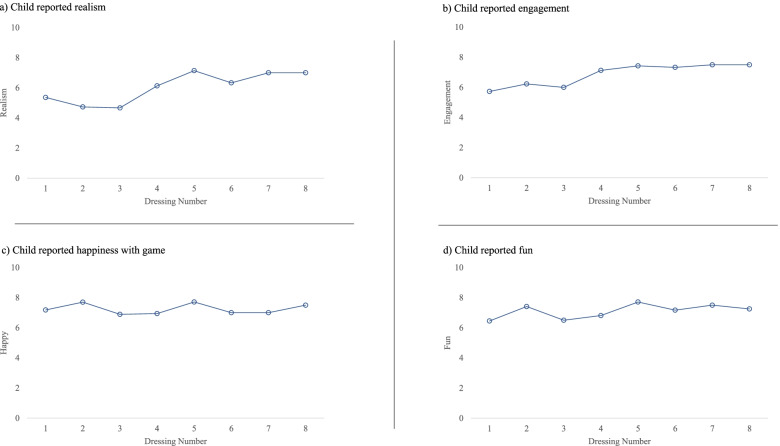
Child reported satisfaction with VR-PAT. **a** Realism, **b** engagement, **c** happiness with game, and **d** fun

All children in the VR group reported their satisfaction with the VR-PAT after each dressing (Fig. 5). As dressing changes progressed over time, children reported increased realism (Did you feel like you were inside the game?) and engagement (How engaging did you think the game was?) with the VR-PAT. Both realism and engagement started at a mean of >5 (NRS, 0–10) at the first dressing and increased to >7 at the last dressing. Children’s happiness (Are you happy with the game?) and fun (How much fun did you have with it?) stayed constant at a mean of >6 (NRS, 0–10) across the week of dressings.

Following the week of dressing changes, children in the VR group were asked about their experience using the VR-PAT (Table 4). When asked about what they liked about the VR game, 54.5% liked the game itself, 36.4% liked that it was a distraction, and 27.3% found the VR-PAT to be calming. When asked what they did not like about the VR game, the most common responses were a desire for more levels or goals (36.4%) or nothing (27.3%). Only one child did not understand how to play the game at first. Finally, children were asked if there were any challenges with the VR-PAT and the majority did not express any challenges (54.5%). Of those who did report challenges, there were some technological issues with downloading the game application (18.2%), getting the system set-up (18.2%), and one found the game to be difficult to play (9.1%).

**Table 4:** Child reported VR-PAT utilization experience

	*N* (%)
**What do you like about the VR game?**^a^	
Playing the game	6 (54.5)
Distraction/not thinking about pain	4 (36.4)
Calming	3 (27.3)
**What do you not like about the VR game?**	
Wanted to stop boat or steer	2 (18.2)
Wanted more levels or goals	4 (36.4)
Did not understand how to play at first	1 (9.1)
Wanted to watch dressing	1 (9.1)
Nothing	3 (27.3)
**Challenges faced during VR-PAT Utilization**	
Game was difficult	1 (9.1)
App download challenges	2 (18.2)
Setting-up the system	2 (18.2)
No challenges	6 (54.5)

^a^Percentages do not add up to 100% as subjects answered multiple options

## Discussion

Our study was mostly made up of male, White children with second degree burns, which is consistent with other burns studies [39]. The number of subjects using pain medication for dressing changes was also consistent with our previous research [31]. Interestingly, children using VR-PAT reported slightly more pain than those in the control group at the beginning of the week, but they reported less pain following the 4th dressing while those in the control group stayed fairly consistent. Caregiver-reported pain followed a similar trajectory across both intervention groups. We saw that the % decrease differed in child-reported and caregiver-reported pain between the control group, with the VR group having a clinically meaningful reduction. All subjects did not complete the same number of surveys, so future analyses should control for dressing numbers, injury severity, age, and medication use. We found that composite pain and opioid consumption (PIOC) score will be innovative and useful for analgesic clinical studies and could be further developed by including binary variables [32–35]. We also saw that children in the VR-PAT group did not report decreasing happiness or fun as the week went on and, in fact, reported increased realism and engagement. There has been some concern in the VR research community that the novelty would wear off with increased exposure, but we found the opposite to be true in this study. This is an encouraging and significant finding for the potential effectiveness of using VR as a pain distraction tool for burn injuries, as these typically require more than one painful procedure. Finally, children provided valuable feedback about the usefulness of using VR at home. Subjects enjoyed playing the game and felt that it helped to be distracted from the dressing change. The things subjects did not like are important to know when either designing a VR game or choosing an existing game for this purpose. We chose to design a game that could be easily used across the age spectrum, but we learned that it may have been too simplistic, particularly for older children. Some children also prefer to be involved in the dressing change process, so having an immersive distraction is actually not preferrable to these children (one child reported this desire in our study). Most subjects in our study found the VR-PAT easy to use, but several important challenges were mentioned, particularly related to the technology and downloading of the app. The one person who found the game to be too difficult was one of the youngest participants in our study, further justifying the lower bound of our age range for inclusion. Importantly, no children found the VR-PAT too difficult to use and stopped using because of this.

We encountered several limitations during this randomized controlled trial. First, summers are the time of year where hospitals usually see the highest numbers of burn injuries in our specified age range. We missed the Summer 2019 due to difficulties in setting up the platform that would allow subjects to download the game app, which was not ready until late August 2019. Second, we encountered multiple changes in Apple’s operating platform security surrounding downloading third party apps, which required our RISI team to change how the VR game could be downloaded onto an iPhone. These changes required complicated workarounds that meant we could not recruit iPhone users for periods of time. A consideration for future studies would be to host the game on Apple’s App Store or Android’s Google Play Store which makes downloading apps easier and could circumvent some of these issues. Third, we missed 5 months (March–August) in 2020, which included the summer, due to a pause on in-person recruitment because of COVID-19. Fourth, COVID-19 resulted in institutional changes to in-person research which shifted our study to limit as much patient contact as possible. The best way to do this was to ask participants to mail their surveys back in a pre-paid, self-addressed envelope, and we made three reminder calls to families. Unfortunately, we experienced a higher rate of loss to follow-up (*n*=10) after making this change and we attribute it to families forgetting to mail surveys and United States Postal Service slowdowns during the COVID-19. We believe that future studies should request surveys to be returned in person, allow families to e-mail their survey responses or create an online survey for the family to submit their surveys by computer or smartphone. Fifth, there were more patients receiving either a long-term dressing or no dressing than we expected, which reduced the number of eligible patients. Our outpatient clinic did not have data on this prior to conducting this study, so this is something we have learned to take into consideration for future studies. Finally, this was our first feasibility trial, so we did not originally design our study using frameworks (such as the Theoretical Framework of Acceptability (TFA), Outcomes for Implementation Research, or the Reach, Effectiveness, Adoption, Implementation, and Maintenance (RE-AIM)). These are valuable frameworks to guide the process of evaluation during feasibility studies and will be incorporated in our future feasibility research.

Through these unexpected challenges, our research team gained significant knowledge on how to conduct a home-based VR pain management research study. We plan to expand on this research by conducting a powered, multi-site RCT study to better examine the effectiveness of VR for pain management during burn dressing changes at home.

## Conclusions

Subjects found the VR-PAT to be a useful distraction during home burn care and reported it be easy to implement. In the VR-PAT group, child- and caregiver-reported pain decreased as the week of dressing changes progressed and saw a clinically meaningful reduction in pain (>30%) as compared to the control group. Children playing the VR-PAT reported consistent happiness and fun as the week went on and increased realism and engagement, which indicates these results were not just due to the novel experience of VR-PAT. Our recommendation is that VR should be considered as a nonpharmacologic pain management approach for home burn dressing changes. Future virtual reality studies need to consider technology issues (like changes in smartphone operating systems), reducing loss to follow-up, and time required to recruit subjects.

## Supplementary Information

**Additional file 1.**

## Data Availability

The datasets generated and/or analyzed during the current study are not publicly available due to them containing private health information (PHI) but are available from the corresponding author on reasonable request.

## Abbreviations

ED: Emergency department
VR: Virtual reality
VR-PAT: VR pain alleviation tool
RCT: Randomized clinical trial
ABA: American Burn Association
CONSORT: Consolidated Standards of Reporting Trials
REDCap: Research Electronic Data Capture
NRS: Numerical rating scale
TBSA: Total body surface area
SD: Standard deviation
AUC: Area under the curve
PIOC: Pain and opioid consumption
TFA: Theoretical Framework of Acceptability
RE-AIM: Reach, Effectiveness, Adoption, Implementation, and Maintenance
