# Supplementary material for: Mobile phone virtual reality game for pediatric home burn dressing pain management: a randomized feasibility clinical trial
Source: Pilot Feasibility Stud. 2022 Aug 18;8:186. doi: 10.1186/s40814-022-01150-9 (PMC9386208; doi:10.1186/s40814-022-01150-9)
Supplement: Supplementary file 1 — Additional file 1. [file 40814_2022_1150_MOESM1_ESM.pdf]

CONSORT checklist of information to include when reporting a pilot trial*

Section/topic and item No	Standard checklist item	Extension for pilot trials	Page No where item is reported
**Title and abstract**			
1a	Identification as a randomised trial in the title	Identification as a pilot or feasibility randomised trial in the title	1
1b	Structured summary of trial design, methods, results, and conclusions (for specific guidance see CONSORT for abstracts)	Structured summary of pilot trial design, methods, results, and conclusions (for specific guidance see CONSORT abstract extension for pilot trials)	1-2
**Introduction**			
Background and objectives:			
2a	Scientific background and explanation of rationale	Scientific background and explanation of rationale for future definitive trial, and reasons for randomised pilot trial	3-5
2b	Specific objectives or hypotheses	Specific objectives or research questions for pilot trial	5
**Methods**			
Trial design:			
3a	Description of trial design (such as parallel, factorial) including allocation ratio	Description of pilot trial design (such as parallel, factorial) including allocation ratio	7-8
3b	Important changes to methods after trial commencement (such as eligibility criteria), with reasons	Important changes to methods after pilot trial commencement (such as eligibility criteria), with reasons	15-16
Participants:			
4a	Eligibility criteria for participants		5-6
4b	Settings and locations where the data were collected		6
4c		How participants were identified and consented	5
Interventions:			
5	The interventions for each group with sufficient details to allow replication, including how and when they were actually administered		8-9
Outcomes:			
6a	Completely defined prespecified primary and secondary outcome measures, including how and when they were assessed	Completely defined prespecified assessments or measurements to address each pilot trial objective specified in 2b, including how and when they were assessed	9
6b	Any changes to trial outcomes after the trial commenced, with reasons	Any changes to pilot trial assessments or measurements after the pilot trial commenced, with reasons	N/A
6c		If applicable, prespecified criteria used to judge whether, or how, to proceed with future definitive trial	N/A

Sample size:			
7a	How sample size was determined	Rationale for numbers in the pilot trial	5
7b	When applicable, explanation of any interim analyses and stopping guidelines		N/A
Randomisation:			
Sequence generation:			
8a	Method used to generate the random allocation sequence		8
8b	Type of randomisation; details of any restriction (such as blocking and block size)	Type of randomisation(s); details of any restriction (such as blocking and block size)	8
Allocation concealment mechanism:			
9	Mechanism used to implement the random allocation sequence (such as sequentially numbered containers), describing any steps taken to conceal the sequence until interventions were assigned		8
Implementation:			
10	Who generated the random allocation sequence, enrolled participants, and assigned participants to interventions		8
Blinding:			
11a	If done, who was blinded after assignment to interventions (eg, participants, care providers, those assessing outcomes) and how		N/A
11b	If relevant, description of the similarity of interventions		N/A
Analytical methods:			
12a	Statistical methods used to compare groups for primary and secondary outcomes	Methods used to address each pilot trial objective whether qualitative or quantitative	10
12b	Methods for additional analyses, such as subgroup analyses and adjusted analyses	Not applicable	N/A
**Results**			
Participant flow (a diagram is strongly recommended):			
13a	For each group, the numbers of participants who were randomly assigned, received intended treatment, and were analysed for the primary outcome	For each group, the numbers of participants who were approached and/or assessed for eligibility, randomly assigned, received intended treatment, and were assessed for each objective	7
13b	For each group, losses and exclusions after randomisation, together with reasons		7
Recruitment:			

14a	Dates defining the periods of recruitment and follow-up		5
14b	Why the trial ended or was stopped	Why the pilot trial ended or was stopped	5
Baseline data:			
15	A table showing baseline demographic and clinical characteristics for each group		11
Numbers analysed:			
16	For each group, number of participants (denominator) included in each analysis and whether the analysis was by original assigned groups	For each objective, number of participants (denominator) included in each analysis. If relevant, these numbers should be by randomised group	10-14
Outcomes and estimation:			
17a	For each primary and secondary outcome, results for each group, and the estimated effect size and its precision (such as 95% confidence interval)	For each objective, results including expressions of uncertainty (such as 95% confidence interval) for any estimates. If relevant, these results should be by randomised group	10-14
17b	For binary outcomes, presentation of both absolute and relative effect sizes is recommended	Not applicable	N/A
Ancillary analyses:			
18	Results of any other analyses performed, including subgroup analyses and adjusted analyses, distinguishing prespecified from exploratory	Results of any other analyses performed that could be used to inform the future definitive trial	N/A
Harms:			
19	All important harms or unintended effects in each group (for specific guidance see CONSORT for harms)		N/A
19a		If relevant, other important unintended consequences	N/A
**Discussion**			
Limitations:			
20	Trial limitations, addressing sources of potential bias, imprecision, and, if relevant, multiplicity of analyses	Pilot trial limitations, addressing sources of potential bias and remaining uncertainty about feasibility	15-16
Generalisability:			
21	Generalisability (external validity, applicability) of the trial findings	Generalisability (applicability) of pilot trial methods and findings to future definitive trial and other studies	14-15
Interpretation:			
22	Interpretation consistent with results, balancing benefits and harms, and considering other relevant evidence	Interpretation consistent with pilot trial objectives and findings, balancing potential benefits and harms, and considering other relevant evidence	14-15
22a		Implications for progression from pilot to future definitive trial, including any proposed amendments	16
**Other information**			

Registration:			
23	Registration number and name of trial registry	Registration number for pilot trial and name of trial registry	2
Protocol:			
24	Where the full trial protocol can be accessed, if available	Where the pilot trial protocol can be accessed, if available	N/A
Funding:			
25	Sources of funding and other support (such as supply of drugs), role of funders		17
26		Ethical approval or approval by research review committee, confirmed with reference number	6

*Here a pilot trial means any randomised study conducted in preparation for a future definitive RCT, where the main objective of the pilot trial is to assess feasibility.
